# A Multimodal User Authentication System Using Faces and Gestures

**DOI:** 10.1155/2015/343475

**Published:** 2015-07-13

**Authors:** Hyunsoek Choi, Hyeyoung Park

**Affiliations:** ^1^School of Electrical Engineering and Computer Science, Kyungpook National University, Deagu 702-701, Republic of Korea; ^2^School of Computer Science and Engineering, Kyungpook National University, Deagu 702-701, Republic of Korea

## Abstract

As a novel approach to perform user authentication, we propose a multimodal biometric system that uses faces and gestures obtained from a single vision sensor. Unlike typical multimodal biometric systems using physical information, the proposed system utilizes gesture video signals combined with facial images. Whereas physical information such as face, fingerprints, and iris is fixed and not changeable, behavioral information such as gestures and signatures can be freely changed by the user, similar to a password. Therefore, it can be a countermeasure when the physical information is exposed. We aim to investigate the potential possibility of using gestures as a signal for biometric system and the robustness of the proposed multimodal user authentication system. Through computational experiments on a public database, we confirm that gesture information can help to improve the authentication performance.

## 1. Introduction

With the growing need for secure authentication methods, various biometric signals are being actively studied. One recent trend is the use of multimodal data for achieving high reliability [1–3]. However, in general, multimodal biometric systems require multiple sensors, which result in high developmental costs. As a new attempt for achieving high reliability and low cost, this paper proposes a novel multimodal biometric system that uses two heterogeneous biometric signals obtained from a single vision sensor: facial image and gesture video.

Face is a representative of physical biometric signals, and many studies have been carried out on developing reliable face recognition systems [4, 5]. However, the performance of face recognition systems is easily influenced by various environmental factors such as illumination, expression, pose, and occlusion. Despite a significant number of studies conducted to overcome these limitations, face recognition systems are still vulnerable and need improvement. Multimodal fusion can be a good solution to overcome this vulnerability [6–8]; however, it incurs a high cost and causes inconvenience. The proposed method is a novel approach to resolve this problem.

Gestures can also be used for user authentication. Gestures are a type of behavioral biometric signals that have recently been considered as good alternatives to physical biometric signals such as faces [9]. The biggest advantage of gestures is changeability by users. Even if physical biometric signals are stolen, users can not change their own physical signal. However, users can change the gesture signals easily like password. Along with the popularization of various IT devices such as smart phones, Kinect, and stereo cameras, a number of studies have been conducted to show that gestures can be used as a good behavioral biometric signal for user authentication. In earlier studies [10–12], it was shown that accelerometer-based gesture recognition is feasible for user authentication in mobile devices. Also, in [13] the accelerometer and the gyroscope on mobile devices were combined for gesture-based user authentication. A novel multitouch gesture-based authentication technique was also proposed [14]. The gesture signal captured by Kinect was also used for user authentication [15, 16]. However, these conventional works require specific sensors such as accelerometer, gyroscope, and depth camera.

Inspired by these previous studies, we propose to use gestures combined with face which can be obtained from a single vision sensor for user authentication. The proposed method can be easily implemented to many types of IT equipment including smart TVs and game devices because it uses only a general vision sensor.

One objective of the proposed method is to show the possibility of gesture video as a biometric signal for user authentication system. Another one is to show the possibility of combining two different biometric signals obtained by a single vision sensor. Although the signals are captured by the same sensor in a single action, they have virtually independent distributional properties, which is desirable for multimodal combination. Therefore, we expect to improve the performance of authentication systems using the proposed combination plan with an insignificant increase in hardware cost. In addition to the benefit of low implementation cost, we take advantage of the common properties of the two different signals. Noting that both face and gesture signals are given as RGB images, we can use common image processing techniques to extract efficient feature matrices from the two signals. Furthermore, we apply an appropriate distance measure to the feature matrices instead of typical distance measures. A comprehensive description of the proposed system and its properties are addressed in the subsequent sections.

## 2. Proposed Multimodal Biometric System

**Figure 1:**
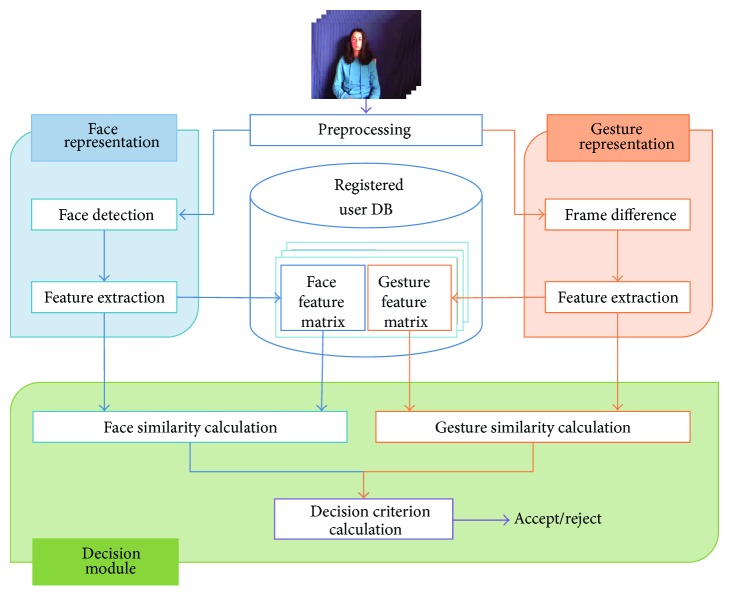
Overall process of the proposed multimodal biometric system, which combines face-based biometrics and gesture-based biometrics.

Figure 1 shows the overall structure of the proposed user authentication system, which is composed of three parts: face representation module, gesture representation module, and decision module. When a video stream that includes face and hand gestures is provided, simple preprocessing such as image resizing and RGB-to-gray transformation is performed. Then, the face and gesture representation modules extract facial and gesture information from the single video and represent each of them using feature matrix, respectively. The decision module uses the two feature matrices to determine whether the given input is authentic or not.

The proposed system operates in two different phases: data registration phase and authentication phase. In the data registration phase, each gallery video is represented by two feature matrices through the face and gesture representation modules, and it is added to user database in the form of two feature matrices. In the authentication phase, a given probe video initially goes through the representation modules to be represented by two feature matrices. Then, the decision module compares the probe feature matrices with the registered gallery feature matrices to determine if the given probe data is authentic or not.

Although detailed description of the representation modules and decision module is given in Sections 3 and 4, respectively, we would like to note a main characteristic of the proposed system. That is, we obtain two biometric signals from a single video stream and use a common feature extraction method for obtaining low-dimensional features from the two signals. This not only reduces the implementation cost but also makes the succeeding process simple. Because the two signals are represented by the same feature descriptor, they can be subjected to the same decision making algorithms.

## 3. Data Representation Modules

### 3.1. Face Representation Module

The face representation module detects a face in a given input video and represents it using a feature matrix. We apply the Viola-Jones face detector [17] to locate the region of the face within an image. It searches for a face in each frame starting with the first frame of the given input video until getting detection results from the face detector.

**Figure 2:**
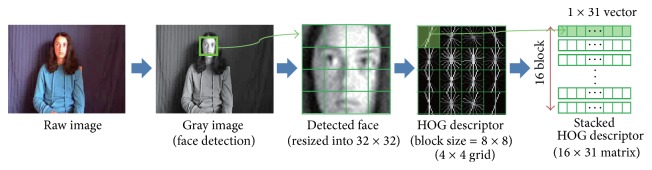
Process of the face representation module.

Once a face is detected, the face area is resized to a 32 × 32 pixel image and we divided face image into a 4 × 4 grid with an 8 × 8 block size for local feature extraction. As a local feature descriptor, we applied a histogram of oriented gradients (HOG) descriptor [18]. We employ the VLFeat library [19] for obtaining a HOG descriptor in implementation. In the VLFeat library, each local grid is represented by 31 dimensional feature vectors so that 16 × 31 feature matrix **F** represents a face.  Figure 2 shows the process of the face representation module.

### 3.2. Gesture Representation Module

In the gesture representation module, frame differencing is initially conducted between two consecutive image frames to capture the area where a gesture movement occurs. It is also possible to eliminate the undesirable effect of the illumination changes and background using frame differencing. Then, we extract the HOG descriptor from each image frame using the same algorithm used in the face representation module. Unlike the face representation module, the difference image is divided into a 6 × 8 grid with a 40 × 40 block size.

**Figure 3:**
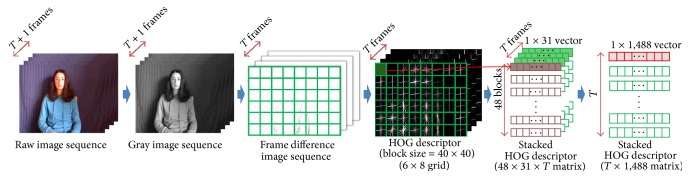
Process of the gesture representation module.

By stacking each HOG feature vector obtained from each difference image row by row, we obtain a *T* × *D* feature matrix **G** for gesture data, where *T* denotes the number of difference images given by a gesture sequence and *D* denotes the dimensionality of the feature vector obtained using the HOG descriptor. Note that *T* varies depending on the length of the input video whereas *D* is fixed (1,488  ( = 6 × 8 × 31) in our actual implementation).  Figure 3 shows the process of the gesture representation module.

## 4. Decision Module and Proposed Similarity Measure

Once a video signal (probe data) is represented by a pair of two feature matrices (**F**_prb_, **G**_prb_), they are used as inputs with user ID and a threshold *θ* for the decision module. At first, the decision module finds a previously registered gallery data (**F**_gal_, **G**_gal_) with given user ID. Then, it calculates distance of faces and gestures, *d*(**F**_prb_, **F**_gal_) and *d*(**G**_prb_, **G**_gal_), respectively. After calculating, the decision module calculates likelihood ratio to determine whether to accept or reject by decision criterion with a threshold *θ*. To achieve a good authentication performance, we focus on two core factors of the decision module: the distance measure and decision criterion.

Note that columns and rows in the face feature matrix **F** and gesture feature matrix **G** have special characteristics. For face feature matrix **F**, each row vector corresponds to local grid in facial image and each column corresponds to a histogram quantity of HOG feature descriptor (see Figure 2). For gesture feature matrix **G**, each row vector corresponds to a frame in gesture video and each column corresponds to a histogram quantity of HOG feature descriptor (see Figure 3). Therefore, typical distance measures for vector data may cause some loss in the relation of time and spatial locality information. We try to maintain the spatial locality of facial image and the sequential relationship between the image frames of the gesture video by using the matrix features directly without vectorization. For this purpose, we employ the matrix correlation distance proposed in our previous works [20] which is a distance measure for matrix data. When two *l*_1_ × *l*_2_ feature matrices **X** and **Y** are given, the matrix correlation distance is defined as

(1) dX,Y=1−ρrowX,Y+ρcolX,Y2,ρrowX,Y=1l1∑i=1l1∑j=1l2xij−mxyij−my∑j=1l2xij−mx2∑j=1l2yij−my2,ρcolX,Y=1l2∑j=1l2∑i=1l1xij−mxyij−my∑i=1l1xij−mx2∑i=1l1yij−my2,

where *m*_*x*_ and *m*_*y*_ are the average of all the elements in **X** and **Y**, respectively. The distance value *d*(**X**, **Y**) is in [0,2], which is similar to the conventional correlation distance. We should note that the distance measure assumes that two matrices **X** and **Y** have the same size. Therefore, in the case of gesture data with various row sizes depending on the length of the video, an additional process is required to perform size alignment of two gesture feature matrices. In this paper, we apply a dynamic time warping (DTW) algorithm [21] to align the rows of matrices, which is a technique to find an optimal alignment between two given sequences.

After computing the distance values *d*_*F*_ = *d*(**F**_prb_, **F**_gal_) and *d*_*G*_ = *d*(**G**_prb_, **G**_gal_), we need to make a decision of acceptance using these values. To do this, we propose a decision criterion based on the likelihood ratio of the distance values, which is defined by

(2) rFGdF,dGpΩA ∣ dF,dGpΩI ∣ dF,dG=pdF,dG ∣ ΩApΩApdF,dG ∣ ΩIpΩI,

where *Ω*_*A*_ denotes the class of distance values from authentic data pairs and *Ω*_*I*_ denotes the class of distance values from impostor data pairs. Therefore, *r*_*FG*_ indicates the ratio of likelihood of whether the distance values (*d*_*F*_, *d*_*G*_) originate from an authentic data pair or an impostor data pair. In other words, a large value of *r*_*FG*_ implies that the observed distance (*d*_*F*_, *d*_*G*_) has a higher possibility of originating from the population of authentic data pairs.

In order to obtain an explicit function for calculating *r*_*FG*_, we need to estimate the probability densities *p*(*Ω*_*A*_∣*d*_*F*_, *d*_*G*_) and *p*(*Ω*_*I*_∣*d*_*F*_, *d*_*G*_). For real world implementation, we assume the Gaussian model for *p*(*d*_*F*_, *d*_*G*_∣*Ω*_*A*_) and *p*(*d*_*F*_, *d*_*G*_∣*Ω*_*I*_) and estimate the parameters using gallery data. Similarly the prior probabilities *p*(*Ω*_*A*_) and *p*(*Ω*_*I*_) are estimated, too. Though the threshold *θ* is set for 1 typically, it is changeable. If *θ* is high, the number of false acceptances is decreased and the number of false rejections is increased. If *θ* is low, the reverse phenomenon occurs. In the experiments, we measure the performance of proposed authentication system with variable *θ*. A summarized description of decision module is presented in Algorithm 1.

## 5. Experimental Results

**Figure 4:**
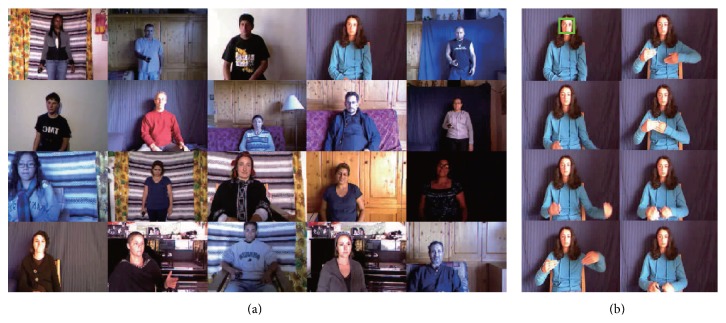
Sample images from ChaLearn database: (a) first frames of 20 selected users, (b) image frames in a gesture video.

In order to confirm the performance of proposed system, we conducted experiments on the ChaLearn database [22], which was built for a gesture recognition competition. Although the data includes depth signals obtained from Kinect, we use only RGB signals because the proposed method is developed for a general vision sensor.  Figure 4 shows some examples of the data. From the whole data set, we prepared three sets—A, B, and C—for experiments. Each set is composed of 80 video samples from 20 subjects; each subject makes his/her own unique gesture four times. Experiments are carried out for each set separately using 4-fold cross-validation. Three samples from each subject are used for gallery data and one sample is used for probe data. Therefore, total 12 experiments were carried out.

Before starting authentication, we first need to estimate two conditional distributions, *p*(*d*_*F*_, *d*_*G*_∣*Ω*_*A*_) and *p*(*d*_*F*_, *d*_*G*_∣*Ω*_*I*_), which are used in decision criterion *r*_*FG*_(*d*_*F*_, *d*_*G*_). For each experiment, we first make all possible data pairs from gallery data and in order to obtain 1,770 distance values, among which 60 values are from authentic pairs and 1,710 from impostor pairs. The estimated pdf *p*(*d*_*F*_, *d*_*G*_∣*Ω*_*A*_) and *p*(*d*_*F*_, *d*_*G*_∣*Ω*_*I*_) using these values are then applied to calculate *r*_*FG*_(*d*_*F*_, *d*_*G*_) in the authentication phase. For evaluating authentication performance, we compute distances between gallery and probe data. Since we have 20 probe samples and 60 gallery samples, there are 1,200 distance values: 60 authentic values and 1,140 impostor values. The performance is evaluated by the error rates (false acceptance and false rejection) of decision module for the 1,200 values.

**Figure 5:**
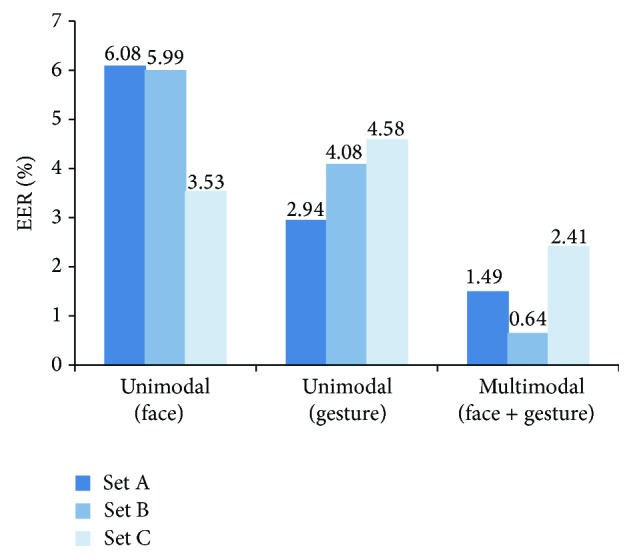
Average EER (%) depending on biosignals using matrix correlation distance.

We compared the performance of the decision module by changing modality and other conventional distance measures. In the unimodal case, we use marginal distribution such as *p*(*d*_*F*_∣*Ω*_*A*_) and *p*(*d*_*G*_∣*Ω*_*A*_) for obtaining the decision criterion. We first compared the value of equal error rate (EER), which is a typical measure for evaluating authentication systems. EER is the value of error rate when the false acceptance rate (FAR) is equal to the false rejection rate (FRR).  Figure 5 shows the average EER over 4-fold cross-validation for each set A, B, and C. As can be seen from Figure 5, gesture-based unimodal system shows slightly better performance than face-based unimodal system. Also, the proposed multimodal biometric system shows the best result.

**Figure 6:**
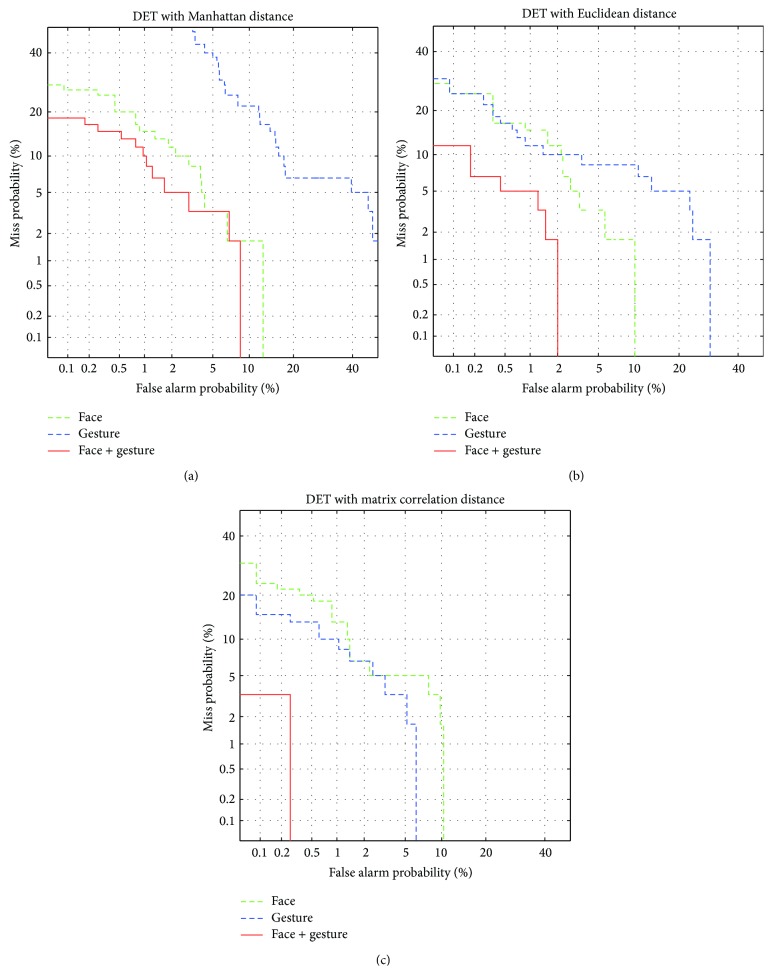
DET curves of authentication system with different modalities: (a) Manhattan distance, (b) Euclidean distance, and (c) matrix correlation distance.

In Figure 6, we present the detection error tradeoff (DET) curves [23] for visualized comparison among different modalities with various distance measures. The DET curve is a plot of error rates for binary classification systems, in which the lower left curve implies the better performance. As can be seen from Figure 6, the proposed multimodal biometric system is superior to unimodal systems regardless of the distance measures. We can also observe that the performance is dependent on the distance measures. For gesture, conventional Manhattan distance and Euclidean distance give poor performance but the matrix correlation distance shows improvement, which is even better than face. This effect is emphasized by the combination of face and gesture, resulting in the remarkable improvement of DET curves as shown in the solid curve of Figure 6(c).

**Figure 7:**
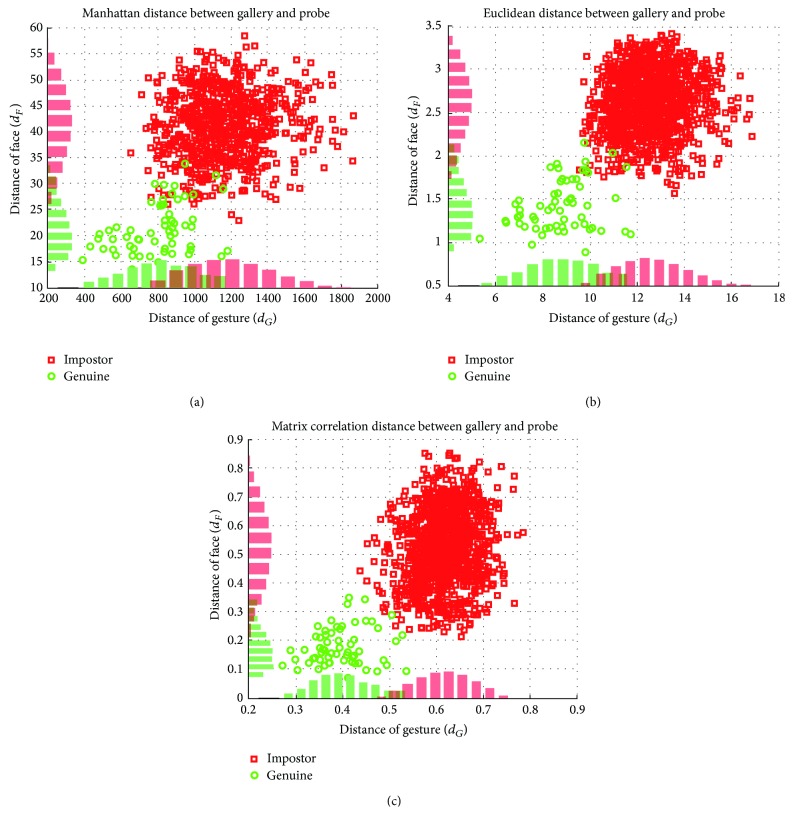
Scatter plots of distance values between authentic pairs (○) as well as impostor pairs (□): (a) Manhattan distance, (b) Euclidean distance, and (c) matrix correlation distance.

Figure 7 shows the scatter plots of the distance values (*d*_*F*_, *d*_*G*_) in *Ω*_*A*_ (○) as well as those in *Ω*_*I*_ (□). In this figure, we can observe that the discriminability is increased by using multimodality. We also plot the marginal histogram of *d*_*F*_ and *d*_*G*_ on the corresponding axes. The overlapped region of histogram implies the region where decision error occurs. In the case of a gesture, we can see that the matrix correlation distance can significantly decrease overlapped region. This means that matrix correlation distance is more appropriate to gesture data with our proposed feature representation. Additionally, we can observe that the bivariate distributions of (*d*_*F*_, *d*_*G*_) have the shape of ellipse, which can justify our Gaussian assumption for estimating the conditional distributions *p*(*d*_*F*_, *d*_*G*_∣*Ω*_*I*_) and *p*(*d*_*F*_, *d*_*G*_∣*Ω*_*A*_). Moreover, from the shape of ellipse, we can guess that the two modalities are almost independent, and this is supported by the fact that the average value of correlation coefficient is 0.19. This property is desirable for combining two biometric signals to construct multimodal biometric system.

## 6. Conclusion

In this paper, we present a look into simple and efficient vision-based multimodal biometric system using heterogeneous biometric signals. By combining physical and behavioral biometric signals, we can achieve a high degree of reliability. Because the proposed system uses a single vision sensor, it can be easily implemented on commonly used smart devices such as smart TVs. More comprehensive study on developing efficient feature extraction and classification will be done for real world application of the proposal system.

## Figures and Tables

**Algorithm 1:**
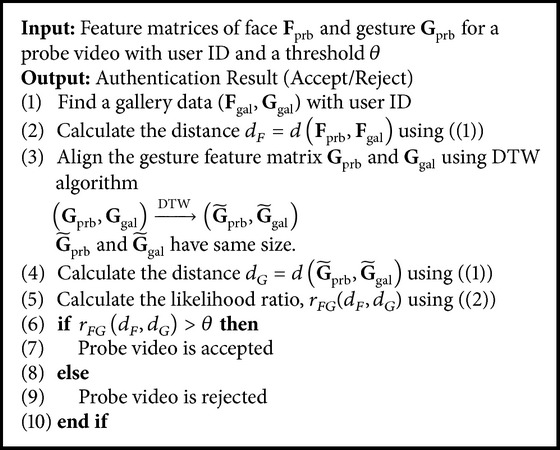
Pseudocode for the decision module.
